# First Experimental Evidence for the Presence of Potentially Virulent *Klebsiella oxytoca* in 14 Species of Commonly Consumed Aquatic Animals, and Phenotyping and Genotyping of *K. oxytoca* Isolates

**DOI:** 10.3390/antibiotics10101235

**Published:** 2021-10-11

**Authors:** Ling Ni, Yingwei Xu, Lanming Chen

**Affiliations:** 1 Key Laboratory of Quality and Safety Risk Assessment for Aquatic Products on Storage and Preservation (Shanghai), Ministry of Agriculture and Rural Affairs of China, College of Food Science and Technology, Shanghai Ocean University, Shanghai 201306, China; lni@shou.edu.cn (L.N.); m190300760@shou.edu.cn (Y.X.); 2 Laboratory of Food Quality and Safety Testing, Shanghai Ocean University, Shanghai 201306, China

**Keywords:** *K*. *oxytoca*, virulence, antibiotic resistance, heavy metal tolerance, genetic diversity, consumable aquatic animals

## Abstract

*Klebsiella oxytoca* is a recently emerging pathogen that can cause necrotizing enterocolitis, hemorrhagic colitis, sepsis-associated purpura fulminans, and infective endocarditis in humans. The bacterium is ubiquitous in water and soil environments. Nevertheless, current literature on *K. oxytoca* in aquatic products is rare. In this study, we surveyed *K. oxytoca* contamination in 41 species of consumable aquatic animals sold in July, August, and September of 2018 and 2019 in Shanghai, China, 40 of which had no history of carrying this bacterium. *K. oxytoca* was for the first time isolated from 14 species with high abundance in benthic animals. None of the *K. oxytoca* isolates (*n* = 125) harbored toxin genes *mviM*, *tisB*, and *yqgB*. However, a high occurrence of virulence-associated genes was observed, including *brkB* (73.6%), *cdcB* (66.4%), *pduV* (64.8%), and *virk* (63.2%). Resistance to sulphamethoxazole-trimethoprim (56.0%) was the most predominant among the isolates, followed by chloramphenicol (6.4%), tetracycline (5.6%), and kanamycin (3.2%). Approximately 8.0% of the isolates displayed multidrug resistant phenotypes. Meanwhile, high percentages of the isolates tolerated the heavy metals Cu^2+^ (84.8%), Pb^2+^ (80.8%), Cr^3+^ (66.4%), Zn^2+^ (66.4%), and Hg^2+^ (49.6%). Different virulence and resistance profiles were observed among *K. oxytoca* isolates in 3 types and 14 species of aquatic animals. The ERIC-PCR-based genome fingerprinting of the 125 *K. oxytoca* isolates revealed 108 ERIC genotypes with 79 singletons, which demonstrated the genetic diversity of the isolates. The results of this study fill gaps for policy and research in the risk assessment of *K. oxytoca* in consumable aquatic animals.

## 1. Introduction

*Klebsiella oxytoca* is a Gram-negative bacterium that ubiquitously resides in water and soil environments [1,2]. The bacterium is also found in the nasopharynx and intestine of healthy individuals in humans [3]. *K.* *oxytoca* was originally isolated from a yogurt specimen in 1886 and named *Bacillus oxytoca*. In 1963, this organism was taxonomically classified as a member of the genus *Klebsiella* [4]. Recently, it has been reported that *K. oxytoca* is the causative agent of necrotizing enterocolitis [5], hemorrhagic colitis [6], sepsis-associated purpura fulminans [7], and infective endocarditis in humans [8], arguing that *K. oxytoca* is an emerging pathogen [1].

Outbreak of *K. oxytoca* in hospitals involved strains with extended-spectrum beta-lactamases and carbapenemases, which consequently lead to therapeutic problems [9]. Multidrug resistant (MDR) *K. oxytoca* isolates of clinical origins have been reported, particularly in developing nations. For instance, Gunduz et al. isolated *K. oxytoca* strains (*n* = 33) from different clinical samples collected in the Department of Pediatrics of Turgut Ozal University in Ankara, Turkey from September 2014 to April 2016. High percentages of these *K. oxytoca* strains were resistant to ciprofloxacin (CIP) (81%), ceftazidime (CAZ) (72%), amikacin (AK) (71%), gentamicin (GEN) (71%), and imipenem (IPM) (59%) [10]. Recently, Alemayehu et al. reported that *K. oxytoca* strains (*n* = 9) isolated from clinical specimens in Sidama, Ethiopia from February 13 to June 7 of 2020 displayed resistance to ampicillin (AMP) (100%), CAZ (100%), CIP (100%), cefuroxime (CRX) (100%), cefotaxime (CTX) (100%), meropenem (MER) (11.1%), GEN (77.8%), cotrimoxazole (COT) (71.4%), nitrofurantoin (NIT) (66.7%), and piperacillin-tazobactam (PIT) (42.9%) [11]. Nevertheless, the available literature on *K. oxytoca* isolates of environmental origins (e.g., aquatic environments and animals) is rare. To our knowledge, few studies have been conducted in this research field. Mann et al. reported on one *K. oxytoca* strain, isolated from a wastewater treatment plant in the North West Province of South Africa, showing resistance to oxytetracycline (OT) [12]. Recently, Håkonsholm et al. collected 476 batches of marine bivalve mollusks along the Norwegian coast from September 2019 to March 2020, including *Mytilus edulis*, *Crassostrea gigas*, *Pecten maximus*, *Modiolus modiolus*, *Arctica islandica*, *Cerastoderma edule*, *Politapes rhomboides*, *Mya arenaria*, and *Strongylocentrotus droebachiensis*. A total of 41 *K. oxytoca* strains were isolated from *M. edulis* and *C. gigas*, 39 of which were resistant to AMP [2]. The presence of *K. oxytoca* in aquatic products poses an emergent threat to food safety systems and the public health.

The pollution of aquatic environments by toxic heavy metals has also given rise to one of the most important ecological and organismic problems [13,14]. The heavy metals accumulated in consumable aquatic animals may lead to serious hazards to humans through the food chain include cadmium (Cd), chromium (Cr), mercury (Hg), nickel (Ni), and lead (Pb) [15]. They increase the selection of antibiotic resistance of bacteria to a certain extent, and vice versa [16]. Heavy metals in the water, in the sediments of rivers, lakes, and oceans, and in fish farming environments worldwide have been reported, particularly in developing countries [17]. For example, Ni et al. recently reported higher positive sample rates (PSR) of the heavy metals copper (Cu) (100%), Hg (100%), Pb (77.4%), and Cd (34.0%) in aquatic product samples (*n* = 108) collected in Shanghai, China in the summer of 2018 and 2019, none of which exceeded individual maximum residue limits (MRLs) [18]. Notably, Alboghobeish et al. reported one *K. oxytoca* strain, ATHA6, isolated from industrial wastewater of Isfahan in Iran, showing resistance to Ni^2+^ (24 mM) [19].

China is the largest producer, consumer, and exporter of aquatic products in the world, accounting for about 60% (65,490,200 tons) of the global amount in 2020 (National Bureau of Statistics, http://www.stats.gov.cn/, accessed on 20 August 2021). In the present study, we conducted a survey on a larger scale to investigate *K. oxytoca* contamination in 41 species of commonly consumed aquatic animals, including 21 species of mollusks, 17 species of fish, and 3 species of crustaceans (Supplementary Material: Table S1). The samples were collected from two large fish markets in July, August, and September of 2018 and 2019 in Shanghai [18], the largest city for aquatic product transportation and consumption in China. To our knowledge, *K. oxytoca* has not ever been detected in these 40 species of aquatic animals. The results in this study provide data to fill gaps for the risk assessment of *K. oxytoca* in aquatic products.

## 2. Results

### 2.1. Prevalence of K. oxytoca in 41 Species of Consumable Aquatic Animals

A total of 567 red, viscous, and single colonies grown on selective MacConkey Inositol Adonitol Carbenicillin (MIAC) agar plates were randomly picked out, which were recovered from 14 of the 41 species of consumable aquatic animals. Approximately 22.0% (125 of the 567 colonies) were identified as *K. oxytoca* by matrix-assisted laser desorption/ionization time of flight mass spectrometry (MALDI-TOF/MS) analysis. Moreover, the 125 *K. oxytoca* strains were detected positive in the capsular staining but negative in Gram’s staining and the dynamic tests. The results were confirmed by 16S rRNA gene sequencing and analysis. Among the 125 *K. oxytoca* isolates, approximately 88.0% (*n* = 110), 11.2% (*n* = 14), and 0.80% (*n* = 1) were recovered from the mollusk, fish, and crustacean samples, respectively (Table S1). Approximately 77.6% (*n* = 97) and 22.4% (*n* = 28) of the isolates were derived from 11 and 3 species of seawater and freshwater animal samples, respectively. Remarkably, most (96.8%, *n* = 121) of the *K. oxytoca* isolates were recovered from benthic aquatic animals.

*K. oxytoca* was present in 14 species of aquatic animals, including 10 species of mollusks: *Anodonta woodiana* (*n* = 21), *Babylonia areolata* (*n* = 5), *Cipangopaludina cahayensis* (*n* = 1), *Haliotis rubra* (*n* = 10), *Mactra antiquata* (*n* = 1), *Mytilus edulis* (*n* = 2), *Neptunea cumingi Crosse* (*n* = 31), *Scapharca subcrenata* (*n* = 5), *Sinonovacula constricta* (*n* = 6), *Tegillarca granosa* (*n* = 28); 3 species of fish: *Blotchy rock cod*, *Carassius auratus* (Crucian)*,* and *Carassius auratus* (Ditrema temmincki Bleeker); and one species of crustacean: *Procambarus clarkii*. Additionally, the other 27 species of aquatic animals were absent from *K. oxytoca* (Table S1).

The 125 *K. oxytoca* isolates were recovered from aquatic animal products originating from four provinces and one city located along the East China Sea, one of the major fishing grounds along China’s coast, which encompasses the Fujian, Jiangsu, Shandong, and Zhejiang provinces, as well as Shanghai City in China. The highest abundance of *K. oxytoca* isolates were observed in the samples originating from Zhejiang Province (32.8%, 41 of the 125 isolates), followed by Fujian Province (25.6%, 32/125), Shandong Province (20.8%, 26/125), and Shanghai City (19.2, 24/125). Only a few isolates (1.6%, 2/125) originated from Jiangsu Province. Approximately 91.2% of the 125 *K. oxytoca* were isolated from the samples collected in the Luchao Port Aquatic Market, and 8.8% from the Jiangyang Aquatic Market in Shanghai, China.

### 2.2. Virulence Associated-Genes in the K. oxytoca Isolates

All of the 125 *K. oxytoca* isolates were detected negative for virulence-associated genes *mviM*, *tisB*, and *yqgB* by PCR assay. Nevertheless, higher incidence of the genes encoding virulence determinants *brkB* (73.6%), *cdcB* (66.4%), *pduV* (64.8%), and *virk* (63.2%) were observed among the isolates. Moreover, *relE* (43.2%), *symE* (37.6%), and *vagC* (23.2%) genes were also detected positive among the isolates. These amplified genes were confirmed by DNA sequencing and analysis, and the obtained sequences were deposited in the GenBank database under the accession numbers MW380322 to MW380328 (Table S2).

The *K. oxytoca* isolates recovered from the three types of aquatic products had different virulence-associated gene profiles. Notably, high percentages of the *brkB*, *cdcB*, *pduV* genes were observed in the *K. oxytoca* isolates recovered from the mollusk (72.7–64.5%) and fish samples (78.6–57.1%). Meanwhile, relatively higher detection frequencies of the *relE*, and *symE* genes were found in the mollusks (44.5%, and 40.9%) than in the fish (28.6%, and 7.1%). The *vagC* and *virk* genes were only present in the isolates recovered from these two types of aquatic products, but with higher incidence from the mollusks (23.6%, 70.0%) than in the fish (21.4%, 14.3%). Additionally, the isolate (*K. o- P. clarkii* 8-1-12-7) from the crustacean (*P. clarkia*) carried the *brkB*, *cdcB*, *relE*, *pduV,* and *symE* genes (Figure not shown).

**Figure 1:**
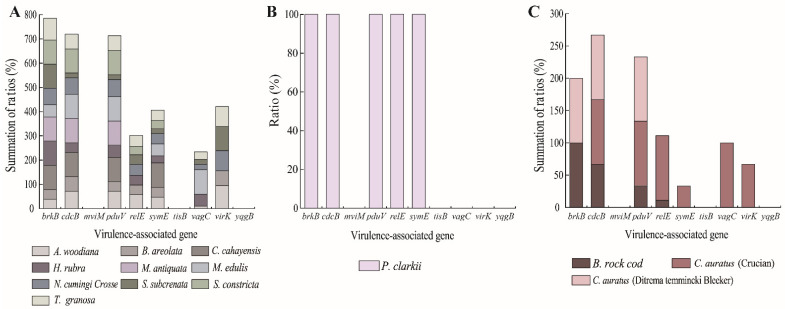
Virulence-related genes of the 125 *K. oxytoca* isolates recovered from 14 species of aquatic animals. (**A**) mollusks; (**B**) crustacean; (**C**) fish.

Based on the limited numbers of *K. oxytoca* isolates recovered from the 14 species of aquatic animals, different virulence-associated gene profiles were also observed (Figure 1A–C). For example, six isolates originated from *A. woodiana*, *N. cumingi* *Crosse*, *S. subcrenata*, and *T. granosa* had the maximum number (*n* = 7, *brkB*^+^/*cdcB*^+^/*pduV*^+^/*relE*^+^/*symE*^+^/*vagC*^+^/*virk*^+^) of the virulence-associated genes tested. Conversely, 17 isolates from the 14 species of aquatic animals carried the smallest number of these genes (*n* = 1, *brkB*^+^ or *virk*^+^).

The 125 *K. oxytoca* isolates harbored 41 different virulence gene profiles (Table S3). Among these, the c*dcB^+^*/*pduV^+^*/*relE^+^*/*symE^+^*/*virk^+^* profile was the most predominant (11.2%, *n* = 14), followed by *BrkB^+^/Virk^+^* (10.4%, *n* = 13), and *BrkB*^+^ (9.6%, *n* = 12). In contrast, one isolate (*K. o*- *A. woodiana* 8-1-8-11) did not harbor any of the virulence-associated genes tested (Table S3).

### 2.3. Antibiotic Resistance of the K. oxytoca Isolates

**Figure 2:**
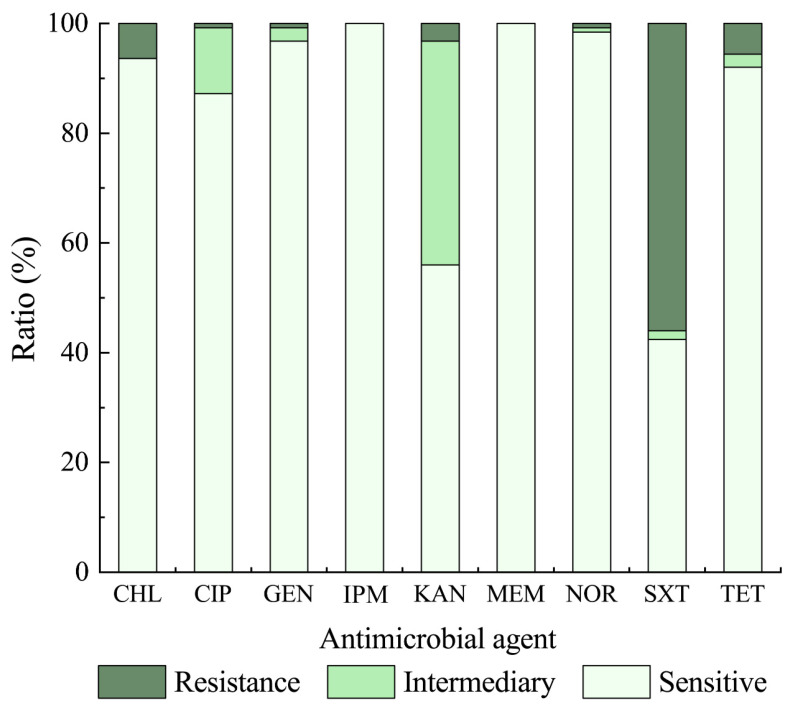
Antimicrobial susceptibility of the 125 *K. oxytoca* isolates.

The susceptibility of the 125 *K. oxytoca* isolates to nine commonly used antibiotics was determined, and the resulting data are illustrated in Figure 2. Approximately 28% of the *K. oxytoca* isolates were susceptible to all the antibiotics evaluated. Moreover, all the isolates were sensitive to imipenem (IPM) (100%) and meropenem (MEM) (100%), and most isolates were also sensitive to norfloxacin (NOR) (98.4%), GEN (96.8%), chloramphenicol (CHL) (93.6%), tetracycline (TET) (92.0%), CIP (87.2%) and kanamycin (KAN) (56.0%). Conversely, sulphamethoxazole-trimethoprim (SXT) resistance was the most predominant (56.0%) among the *K. oxytoca* isolates, followed by CHL (6.4%), TET (5.6%), and KAN (3.2%). Approximately 12.0% and 40.8% of the isolates also exhibited intermediate susceptibility to CIP and KAN, respectively. The resistance trend of the 125 *K. oxytoca* was SXT > CHL > TET > KAN > CIP = GEN = NOR > MEM = IPM (Figure 2).

The *K. oxytoca* isolates recovered from the 3 types of aquatic products had different antibiotic resistance profiles (Figure not shown). Higher rates of resistance to SXT were observed in the isolates recovered from the mollusks (60.9%, 67/110) than those from the fish (21.4%, 3/14) and crustacean (0%, *n* = 1). The CHL, TET, KAN, and GEN resistance were solely present in the isolates from the mollusks (7.3%, 6.4%, 3.6%, 0.9%), whereas CIP and NOR resistance were only found in the isolates derived from the fish (7.1%, and 7.1%) (Figure not shown).

**Figure 3:**
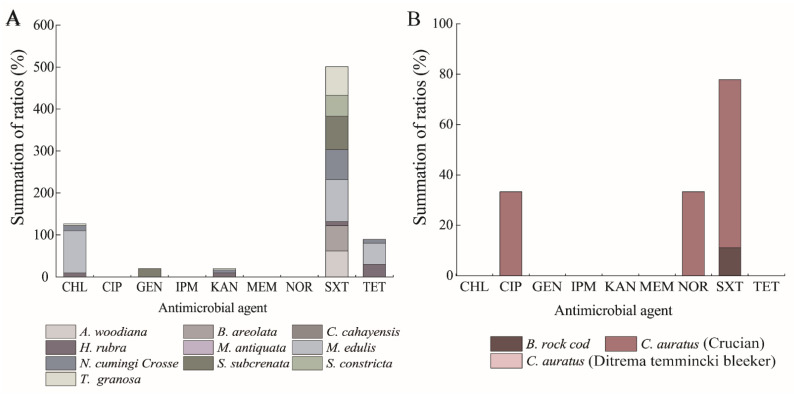
Antibiotic resistance of the 125 *K. oxytoca* isolates in the 13 species of aquatic animals. (**A**) mollusks; (**B**) fish.

Additionally, different antibiotic resistance profiles were also observed among the *K. oxytoca* isolates recovered from the 14 species of aquatic animals (Figure 3A,B). Remarkably, resistance to SXT was prevalent among the isolates from 10 of the 14 species, except *C. cahayensis*, *C. auratus* (Ditrema temmincki Bleeker), *M. antiquate*, and *P. clarkii.* For example, all isolates (*n* = 2) recovered from *M. edulis* were resistant to SXT, and more than half of the isolates from *A. woodiana* (61.9%, 13/21), *B. areolate* (60%, 3/5), and *S. constricta* (50%, 3/6) showed resistance to SXT. Resistance to CHL was present in a few isolates originating from *H. rubra* (1 of 10 isolates), *M. edulis* (2 of 2 isolates)*, N. cumingi Crosse* (4 of 31 isolates), and *T. granosa* (1 of 28 isolates). One isolate (*K. o*- *C. auratus* 8-11-1) from *C. auratus* (Crucian) showed resistance to CIP and NOR, while GEN resistance was solely observed in one isolate (*K. o*- *S. subcrenata* 8-2-11) from *S. subcrenata*. Additionally, none of the isolates from *C. cahayensis* (*n* = 1), *C. auratus* (Ditrema temmincki Bleeker) (*n* = 2), *M. antiquata* (*n* = 1), and the crustacean *P. clarkii* (*n* = 1) was resistant to any of the nine antibiotics evaluated in this study.

### 2.4. MDR Phenotypes of the K. oxytoca Isolates

Approximately 8.0% (*n* = 10) of the *K. oxytoca* isolates had MDR phenotypes showing resistance to two or more antimicrobial agents. The isolates originating from the mollusks had the highest occurrence of MDR phenotypes (8.2%, 9/110), followed by the fish (7.1%, 1/14), and the crustacean isolates (0.0%, 0/1). Moreover, the MDR isolates were recovered from 6 of the 14 species of aquatic animals, including *M. edulis* (*n* = 2), *C. auratus* (Crucian) (*n* =1), *S. subcrenata* (*n* = 1), *N. cumingi Crosse* (*n* = 4), *H. rubra* (*n* = 1), and *T. granosa* (*n* = 1). Conversely, no MDR strain was found in the other 8 species of aquatic animals.

The multiple antimicrobial resistance index (MARI) values of the 125 *K. oxytoca* isolates ranged from 0.44 to 0.00, which indicated varying degrees of exposure to the nine antibiotics evaluated. The mean MARI values of the isolates originating from the mollusks, fish, and crustacean were 0.21, 0.15, and 0, respectively. Among the 14 species of aquatic animals, the maximum MARI value was found from the isolates recovered from *N. cumingi Crosse* (0.44), and *H. rubra* (0.44), followed by *C. auratus* (Crucian) (0.33), *M. edulis* (0.33), *T. granosa* (0.33), and *S. subcrenata* (0.22), whereas the isolates from the other 8 species had smaller MARI values (≤0.11). Notably, two isolates (*K. o*- *H. rubra* 8-2-2-11 and *K. o*- *N. cumingi Crosse* 8-6-19), originating from *H. rubra* and *N. cumingi Crosse*, respectively, had the largest MARI value of 0.44, showing resistance to four of the nine antibiotics tested.

### 2.5. Heavy Metal Tolerance of the K. oxytoca Isolates

Tolerance of the 125 *K. oxytoca* isolates to 8 heavy metals was determined (Table S4). The maximum observed values of minimal inhibitory concentrations (MICs) among the *K. oxytoca* isolates were 3200 μg/mL for Cr^3+^, Pb^2+^, Mn^2+^, and Cu^2+^; 1600 μg/mL for Ni^2+^, and Zn^2+^; 800 μg/mL for Cd^2+^; and 50 μg/mL for Hg^2+^, when compared with the quality control strain *E. coli* K12. Tolerance to Cu^2+^ and Pb^2+^ were most prevalent among the isolates (84.8%, 80.8%), followed by Cr^3+^ (66.4%), Zn^2+^ (66.4%), Hg^2+^ (49.6%), Mn^2+^ (11.2%), Cd^2+^ (9.6%), and Ni^2+^ (0.8%). The tolerance trend of the 125 *K. oxytoca* isolates to heavy metals was Cu^2+^ > Pb^2+^ > Cr^3+^ = Zn^2+^ > Hg^2+^ > Cd^2+^ > Mn^2+^ > Ni^2+^.

*K. oxytoca* isolates recovered from the three types of aquatic products had different heavy metal tolerance profiles. The majority of the isolates from all three types of aquatic products were tolerant to Cu^2+^ (100% to 84.5%), Hg^2+^ (100% to 48.2%), Pb^2+^ (100% to 79.1%) and Zn^2+^ (100% to 61.8%). Tolerance to Cr^3+^ was prevalent among the isolates from the fish (100%) and mollusks (62.7%), whereas lower percentages of Cd^2+^ tolerance were found in the isolates from the mollusks (10.0%) and fish (7.1%). Tolerance to Mn^2+^ (12.7%) and Ni^2+^ (0.9%) were solely observed in the isolate of the mollusks (Figure not shown).

**Figure 4:**
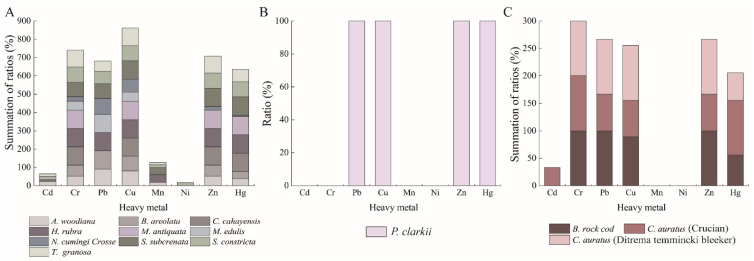
Heavy metal tolerance profiles of the 125 *K. oxytoca* isolates recovered from the14 species of aquatic animals. (**A**) mollusks; (**B**) crustacean; (**C**) fish.

Different heavy metal tolerance profiles were also found among the *K. oxytoca* isolates in the 14 species of aquatic animals (Figure 4A–C). The *K. oxytoca* isolates recovered from *S. constricta* were tolerant to all eight of the heavy metals tested, followed by *T. granosa*, and *H. rubra* (7 heavy metals); *S. subcrenata,* and *C. auratus* (Crucian) (6 heavy metals); *C. auratus* (Ditrema temmincki Bleeker), *B. areolata*, *B. rock cod*, and *N. cumingi Crosse* (5 heavy metals). Tolerance to Ni^2+^ was only found in one isolate from *S. constricta* (16.7%, 1/6).

### 2.6. Genetic Diversity of the K. oxytoca Isolates

**Figure 5:**
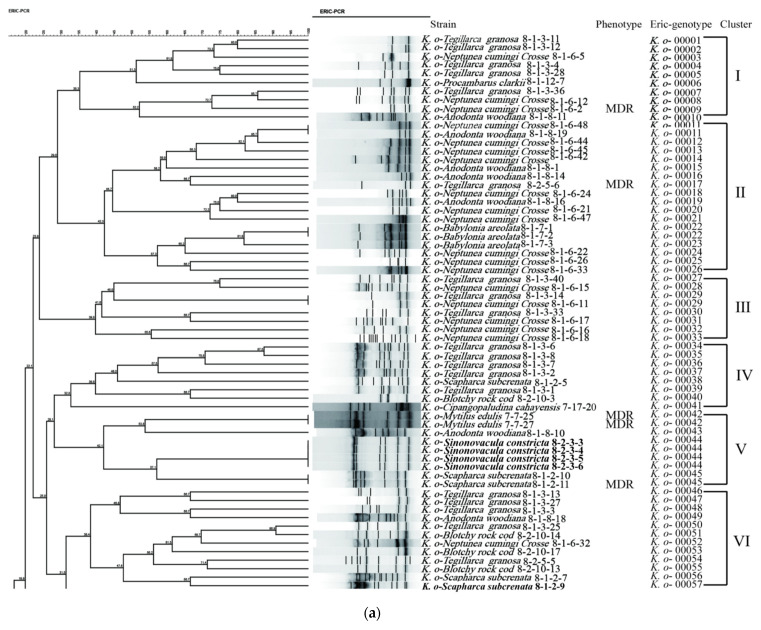

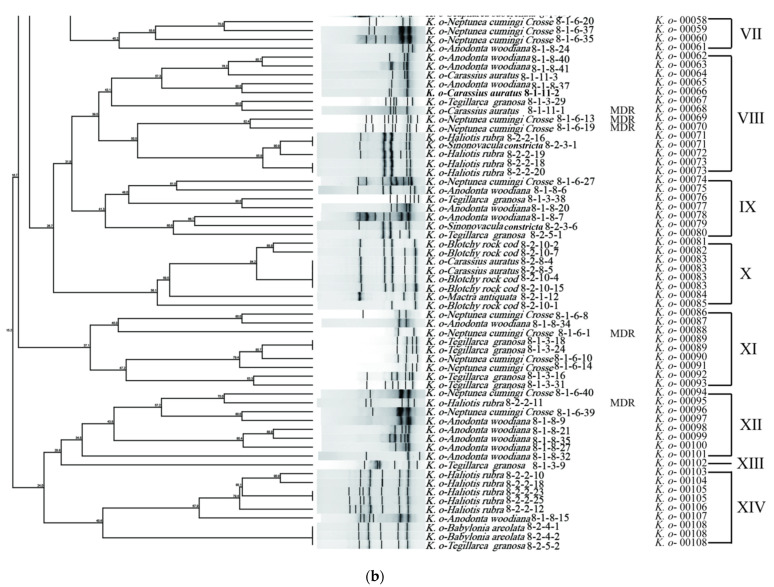
ERIC-PCR-based genotyping of the 125 *K. oxytoca* isolates.

The enterobacterial repetitive intergenic consensus-PCR (ERIC-PCR) was used to analyze the genetic diversity of the 125 *K. oxytoca* isolates recovered from the 14 species of aquatic animals. The obtained genome fingerprinting profiles comprised various numbers of DNA bands mainly ranging from 100 to 1000 bp (Figure 5a,b). Based on the fingerprinting profiles, all the isolates were classified into 108 different ERIC-genotypes, 73.2% of which were assigned as singletons (*n* = 79). Approximately 36.7% (*n* = 29), 30.4% (*n* = 24), and 25.3% (*n* = 20) of these singletons were derived from the mollusks *N. cumingi Crosse*, *T. granosa*, and *A. woodiana*, respectively. The UPGMA algorithm grouped all the 108 ERIC genotypes into 14 distinct clusters (clusters I to XIV) at a 32.0% similarity cut-off level (Figure 5). Approximately 14.4% (*n* = 18) of the *K. oxytoca* isolates were classified into the largest cluster, II, followed by 11.2% (*n* = 14), 9.6% (*n* = 12), and 8.0% (*n* = 10) into clusters VIII, III, and I, respectively. The remaining isolates (56.8%) fell into clusters III–V, VII, and IX–XIV (7.2% to 0.8%). Most isolates had a similarity coefficient of 30.0–85.0%, and a Simpson’s diversity index of 0.8485. These results demonstrate the considerable genetic diversity of the 125 *K. oxytoca* isolates recovered from the 14 species of aquatic animals.

Approximately 23.2% (*n* = 29) of the 125 *K. oxytoca* isolates shared 12 ERIC-genotypes. For example, four isolates with the ERIC-genotype *K. o*- 00044 were derived from *S. constricta*, suggesting near-present relatives or clonal relatedness. Additionally, there were four ERIC-genotypes (*K. o*- 00011, *K. o*- 00071, *K. o*- 00083, and *K. o*- 00108) containing the isolates derived from different species of aquatic animals. For example, the two isolates *K. o*-*H.* *rubra 8*-2-2-16, and *K. o*- *S. constricta* 8-2-3-1, which share the same ERIC-genotype *K. o*- 00071, were recovered from *H.* *rubra* and *S. constricta*, respectively.

### 2.7. Comparison of the MDR and Heavy Metal Tolerance

**Figure 6:**
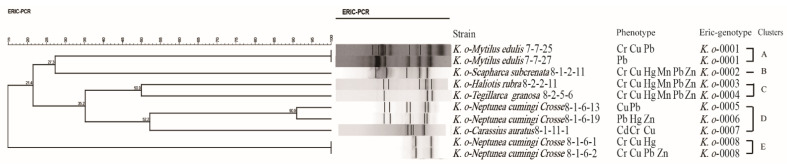
ERIC-PCR-based genotyping of the 10 *K. oxytoca* isolates with MDR phenotypes.

To get insights into co-selection between antibiotics and heavy metals in *K. oxytoca*, we further analyzed the 10 isolates with MDR phenotypes by phylogenetic analysis (Figure 6). The resulting data revealed the genetic diversity of the MDR *K. oxytoca* isolates with a Simpson’s index of 0.8556. Moreover, the MDR isolates belonging to 8 ERIC-genotypes were classified into 5 distinct clusters (clusters A–E) (Figure 6).

Most of the MDR *K. oxytoca* isolates (90.0%, *n* = 9) were tolerant to two or more heavy metals. Specifically, one *K. oxytoca* isolate (*K. o*- *N. cumingi crosse* 8-1-6-19) displayed resistance to 4 antibiotics (CHL/SXT/KAN/TET), and was also tolerant to 3 heavy metals (Pb^2+^/Hg^2+^/Zn^2+^). Another *K. oxytoca* isolate (*K. o*- *H. rubra* 8-2-2-11) was resistant to 4 antibiotics (CHL/SXT/KAN/TET) and 6 heavy metals (Cr^3+^/Cu^2+^/Hg^2^/Mn^2+^/Pb^2+^/Zn^2+^). These results provided direct evidence for the cross-resistance between the MDR and heavy metals in the *K. oxytoca* isolates originating from consumable aquatic animals.

## 3. Discussion

*K. oxytoca* is the second most common *Klebsiella* species after *K. pneumoniae* [5]. Nevertheless, systematic research on *K. oxytoca* is still in its infancy, and current literature on *K. oxytoca* in aquatic products is rare [1,20]. Recently, Håkonsholm et al. reported 40 *K. oxytoca* strains isolated from *M. edulis* and one from *C. gigas* [2]. In this study, we surveyed *K. oxytoca* contamination in 41 species of commonly consumed aquatic animal products sold in Shanghai in July, August, and September of 2018 and 2019. *K. oxytoca* was for the first time isolated from 14 species of aquatic animals, including 10 species of mollusks: *A. woodiana*, *B. areolata*, *C. cahayensis*, *H. rubra*, *M. antiquata*, *M. edulis*, *N. cumingi* *Crosse*, *S. subcrenata*, *S. constricta* and *T. granosa*; 3 species of fish: *B. rock cod*, *C. auratus* (Crucian), and *C. auratus* (Ditrema temmincki Bleeker); and one species of crustacean: *P. clarkii*. Our data also provided the first experimental evidence for high detection frequencies of *K. oxytoca* in benthic aquatic animals, such as *N. cumingi crosse*, *T. granosa*, and *A. woodiana*. These results suggested a potential health risk of the bacterial transmission to communities through the aquatic animals.

The cytotoxin-producing *K. oxytoca* has recently been identified as a new candidate etiologic agent in the pathogenesis of necrotizing enterocolitis [5]. In fact, information in genes associated with the virulence of *K. oxytoca* still remains to be discovered. Previous studies have indicated that the genes encoding adhesins, siderophores, and invasins of the genus *Klebsiella* were associated with deleterious traits [21]. In this study, virulence-related genes (*brkB*, *cdcB*, *pduV*, *relE*, *symE*, *vagC*, and *virK*) in the 125 *K. oxytoca* isolates recovered from 14 species of aquatic animals were detected simultaneously. High incidence of the *brkB* (73.6%), *cdcB* (66.4%), *pduV* (64.8%), and *virk* (63.2%) genes was observed. The *brkB* gene encodes the YihY/virulence factor BrkB family protein, while *cdcB*, *pduV*, and *virK* encode a cytotoxin, a type I toxin-antitoxin system hok family toxin, and the virulence factor VirK, respectively [22,23]. The RelE toxin in *Escherichia* *coli* was a global inhibitor of translation [24]. SymE was toxic in the inhibition of protein synthesis and RNA degradation [25]. The virulence factor VagC was involved in the bacteriocin secretion system and the type II toxin-antitoxin system in Turkish *Salmonella* serovar Infantis isolates [26]. In this study, the virulence-associated genes *mviM,* *tisB,* and *yqgB* were absent from the *K. oxytoca* isolates. As a virulence gene, *mviM* was associated with the exercise, biofilm formation and antimicrobial resistance of *Cronobacter sakazakii* [27]. TisB is a component of the TisB/IstR-1 toxin-antitoxin system in *E*. *coli* [28]. The adaptive factor YqgB in *Bacillus thuringiensis* facilitated the bacterial colonization of the host [29]. In this study, the *K. oxytoca* isolates recovered from the 3 types and 14 species of aquatic animals harbored different virulence-associated gene profiles. Remarkably, the isolates originating from *A. woodiana*, *N. cumingi crosse*, *S. subcrenata*, and *T. granosa* harbored the maximum number (*n* = 7) of the virulence-associated genes tested, which suggests a health risk from the potentially virulent *K. oxytoca* in these consumable aquatic animals.

The propagation and spread of resistant pathogenic bacteria pose serious threats to the public heath for humans and animals [30,31]. It is estimated that antibiotic resistance may lead to 10 million deaths per year by 2050 [31]. Misuse of antimicrobial agents is the main cause of antibiotic resistance in pathogenic bacteria, particularly in developing nations [32,33,34]. In this study, our results indicate that SXT resistance was the most predominant (56.0%) among the 125 *K. oxytoca* isolates of aquatic animal origins. SXT-resistant *K. oxytoca* isolates in humans has also been reported. For example, Maharjan et al. recently reported that 9.4% of *K. oxytoca* strains (*n* = 48) isolated from stool specimens of healthy adult volunteers (*n* = 510) in Kathmandu in Nepal showed the highest resistance toward SXT (45.8%) [35]. In this study, incidences of intermediate susceptibility to KAN (40.8%), and CIP (12.0%) were observed, which suggests a potential resistance trend of *K. oxytoca* in aquaculture environments.

MARI is commonly used to determine the health risk associated with antibiotic resistance [17]. In this study, the mean MARI values for *K. oxytoca* isolates derived from the mollusks, fish, and crustacean samples were 0.21, 0.15 and 0, respectively. Among the 14 species of aquatic animals, the maximum MARI value was derived from the isolates in *H. rubra* (0.44), and *N. cumingi Crosse* (0.44). These results suggested likely extended exposure of the mollusks, particularly *H. rubra*, and *N. cumingi Crosse*, to the antibiotic drugs evaluated in this study. Recently, Ni et al. reported that 10 antibiotics (AMP, CHL, CIP, ENR, OFX, OT, pefloxacin (PEF), sulfadiazine (SDZ), sulfisomidine (SIM), and sulfapyridine (SP)) were detected positive in the 41 species of aquatic animal samples (*n* = 108) with an overall detection frequency of 61.3%. The residual CHL (47.2%), AMP (31.1%), and SP (1.9%) exceeded their MRLs [18], which provided direct evidence for the antibiotic-resistant phenotypes of *K. oxytoca* isolates observed in this study.

A high level of the bioaccumulation of toxic heavy metals (e.g., Cd^2^^+^, Cr^3+^, Hg*^2+^*, Ni*^2+^*, and Pb^2+^) through the food chain is a grave threat to human health, due to their non-degradable nature [36]. Numerous studies have reported heavy metal residues in various aquatic environments and species of food animals (shellfish, fish, crustaceans, and crabs) sampled worldwide, particularly in developing nations [13,14,15,18,37]. In the recent surveys by our research group [17,38,39], the heavy metal-tolerant waterborne pathogen *Vibrio cholerae* has been discovered in many species of aquatic animals. In this study, the results indicated that the *K. oxytoca* isolates originated from the 3 types and 14 species of aquatic animals had different heavy metal tolerance profiles. Tolerance to Cu^2+^ and Pb^2+^ was the most prevalent among the isolates (84.8% and 80.8%). Cu is an essential element for various biological functions in many organisms, and it is also a cofactor for hemocyanin in aquatic arthropods and mollusks. However, excessive levels of Cu can be toxic or disrupting metabolic processes [40]. In this study, our data also revealed a higher detection frequency of Cr^3+^ tolerance in the *K.* *oxytoca* isolates from the fish samples than that from the mollusks. Moreover, the isolates recovered from *S. constricta* were tolerant to all 8 heavy metals, followed by *H. rubra*, and *T. granosa* (7 heavy metals); *C. auratus* (Crucian), and *S. subcrenata* (6 heavy metals); *B. areolata*, *B. rock cod, C. auratus* (Ditrema temmincki Bleeker), and *N. cumingi Crosse* (5 heavy metals). Tolerance to Ni*^2+^* was only detected in the isolates from *S. constricta*. These data suggest serious heavy metal pollution likely occurred in the aquaculture environments, consistent with previous reports [18,38,39]. Recently, Ni et al. reported that the heavy metals Cu, Hg, Pb, and Cd were observed in the 41 species of aquatic animals with PSRs of 100%, 100%, 77.4%, and 34.0%, respectively, none of which exceeded their MRLs [18], which provided direct evidence for the high incidence of *K. oxytoca* tolerance to heavy metals in this study. Additional attention should be paid to the potential health risk of heavy metal pollution in consumable aquatic animals sold in Shanghai, China.

In this study, ten *K. oxytoca* isolates had MDR phenotypes, nine of which also exhibited tolerance to two or more than two heavy metals. For example, the *K. oxytoca* isolate *K. o- H. rubra* 8-2-2-11, showing resistance to the four antibiotics CHL, SXT, KAN, and TET, also tolerated the 6 heavy metals Cr^3+^, Cu^2+^, Hg^2+^, Mn^2+^, Pb^2+^, and Zn^2+^. These results suggested that heavy metal pollution likely co-selected for antibiotic resistance in *K. oxytoca*, and vice versa. In our previous research, antibiotic resistance correlating positively with heavy metal resistance was also observed in the *V. cholerae* isolates recovered from the 41 species of aquatic animals [18]. Cross-resistance due to co-selection can be inferred as the most likely mechanism of the rising antibiotic-resistant pathogens [41].

## 4. Materials and Methods

### 4.1. Sample Collection

Forty-one species of fresh aquatic animals, including 21 species of mollusks, 17 species of fish, and 3 species of crustaceans (Table S1), were sampled from Shanghai Jiangyang Aquatic Market (31°21′25.90″ N, 121°26′50.68″ E), and Shanghai Luchao Port Aquatic Market (30°51′40.21″ N, 121°50′48.40″ E) located in Shanghai, China in July, August, and September of 2018 and 2019 [18]. The samples (*n* = 108) were collected in sterile plastic bags (Nanjing Maojie Microbial Technology Co., Ltd., Nanjing, China), stored in iceboxes (700 × 440 × 390 mm), and immediately transported to the laboratory at Shanghai Ocean University, Shanghai, China for the analyses described below.

### 4.2. Isolation and Identification of K. oxytoca

*K. oxytoca* was isolated and identified in agreement with the instructions of the Chinese Government Standard (SN/T 1962–2007). Briefly, 25 g of each fish intestine sample or whole-tissue mollusk or crustacean sample was mixed with 225 mL sterile 1 × phosphate-buffered saline (PBS, pH 7.4–7.6, Shanghai Sangon Biological Engineering Technology and Services Co., Ltd., Shanghai, China), and then homogenized for 10 min using the Stomacher (Bagmixer 400 W, Interscience, Saint Nom la Bretèche, France). Ten-fold serial dilutions were prepared, and 100 μL of each dilution (to 1:10^4^) was spread onto the selective MIAC (Beijing Land Bridge Technology Co., Ltd., Beijing, China) agar medium, and then incubated at 37 °C for 18 h. The red, wet, and sticky colonies that were 2 to 3 mm in diameter grown on the MIAC agar plates were picked out for further analysis.

Presumptive *K. oxytoca* colonies were identified using the MALDI-TOF/MS platform (Microflex LT/SH, MALDI Biotyper, Bruker, Germany) [42]. Briefly, 1.2 mL of 75% ethanol (Sangon Biotech Co., Ltd., Shanghai, China) and 50 μL of 70% formic acid (Sinopharm Chemical Reagent Co., Ltd., Shanghai, China) were added to the bacterial culture of each strain, according to the protocol recommended by the manufacturer (Bruker Daltonics, Bremen, Germany). The mixture was centrifuged at 14,000 rpm for 2 min and the supernatant was used for MALDI identification. The sample was spotted on the MBT Biotarget 96 target plates. After air-drying, 1 mL of saturated α-cyano-4-hydroxycinnamic acid (HCCA) (Bruker Daltonics, Bremen, Germany) matrix solution in 50% of acetonitrile and 2.5% of trifluoroacetic acid (Sinopharm Chemical Reagent Co., Ltd., Shanghai, China) was added. Mass spectra were acquired using a Microflex LT Mass Spectrometer (Bruker Daltonics, Bremen, Germany) with default parameters, including detection in linear positive mode, laser frequency of 60 Hz, ion source voltages of 2.0 and 1.8 kV, and lens voltage of 6 kV, within the m/z of 2000–20,000. For each strain, a total of 24 spectra from eight independent spots were acquired. External calibration of the mass spectra was performed using the Bruker bacterial test standard (BTS, Bruker Daltonics, Bremen, Germany) [42].

*K. oxytoca* strains were further identified by biochemical tests, including the capsular staining, Gram′s staining, and dynamic tests, using biochemical identification reagents (Qingdao haibo Bio Co., Ltd., Qingdao, China) following the instructions of the manufacturer and the Chinese Government Standard (SN/T 1962–2007). *K. oxytoca* strains were also identified by 16S rRNA gene amplification and sequencing using the universal bacterial primers 27F and 1492R (Table S5) as described previously [43]. Standard strain *K. oxytoca* ATCC43165 (Guangdong Culture Collection Center, Guangzhou, China) was used as a positive control strain in this study.

### 4.3. Detection of Virulence Genes in K. oxytoca

Virulence-associated genes (*brkB,* *cdcB,* *mviM,* *pduV,* *relE,* *symE,* *tisB,* *vagC,* *virK,* *yqgB*) were detected by PCR assay. All oligonucleotide primers (Table S5) were synthesized by Shanghai Sangon Biological Engineering Technology and Services Co., Ltd., Shanghai, China. PCR products were purified using Takara MiniBEST DNA Fragment Purification Kit Ver. 4.0 (Takara Biomedical Technology (Beijing) Co., Ltd., Beijing, China). Genomic DNA was extracted using TaKaRa MiniBEST Bacterial Genomic DNA Extraction Kit Ver.3.0 (TaKaRa, Beijing, China) following the manufacturer’s instructions. The 20 μL PCR reaction mixture contained 8 μL of DNase/RNase-free deionized water (Tiangen Biotech Co., Ltd., Beijing, China), 10 μL of 2× Taq Master Mix (Novoprotein Technology Co., Ltd., Shanghai, China), 0.5 μL of each primer (5 μM), and 1 μL of DNA template. PCR reactions were performed as described previously [17,38,39], but with different annealing temperatures and elongation times based on the melting temperatures of the primer pairs and the predicted sizes of the PCR products (Table S5). The extracted DNA samples and amplified PCR products were analyzed by agarose gel electrophoresis analysis, visualized, and recorded as described previously [17,38,39]. The PCR products were purified and sequenced by Suzhou Jinweizhi Biotechnology Co., Ltd., Suzhou, China. DNA sequences were analyzed using the basic local alignment search tool (BLAST) of the National Center for Biotechnology Information (NCBI, https://www.ncbi.nlm.nih.gov/, accessed on 19 August 2021).

### 4.4. ERIC-PCR Assay

Genotyping was conducted by ERIC-PCR with the primers ERIC1R and ERIC2 (Table S5) [39,44]. The 20 μL reaction mixture contained 6 μL of DNase/RNase-free deionized water (Tiangen, China), 10 μL of 2 × Taq Master Mix (Novoprotein Technology, Shanghai, China), 1 μL of each primer (5 μM), and 2 μL of a DNA template. ERIC-PCR reactions were performed at 95 °C for 30 s, 52 °C for 1 min, and at 65 °C for 8 min for 32 reaction cycles. Amplified DNA fragments were visualized, recorded, and analyzed using BioNumerics 7.6 software, as described earlier [17,38,39]. Cluster analysis was performed by the unweighted pair group with arithmetic averages (UPGMA), and the Simpson index was calculated for the biodiversity of *K. oxytoca* isolates [17,38,39].

### 4.5. Antibiotic Susceptibility and Heavy Metal Tolerance Assays

The *K. oxytoca* isolates were measured for susceptibility to nine antimicrobial agents (Oxoid, UK) according to the method issued by the Clinical and Laboratory Standards Institute (2018, CLSI M100-S28), for CHL (30 μg), CIP (5 μg), GEN (10 μg), IPM (10 μg), KAN (30 μg), MEM (10 μg), NOR (10 μg), SXT (23.75 μg and 1.25 μg), and TET (30 μg). *K. oxytoca* ATCC43165 was used as a quality control strain. Broth dilution testing (microdilution) was used to measure the MICs of 8 heavy metals, including HgCl_2_, NiCl_2_, CrCl_3_, CdCl_2_, PbCl_2_, CuCl_2_, ZnCl_2_, and MnCl_2_ (Analytical Reagent, Sinopharm Chemical Reagent Co., Ltd., Shanghai, China) [17,38,39]. *E. coli K12* (Institute of Industrial Microbiology, Shanghai, China) was used as a quality control strain. All tests were performed in triplicate in this study.

### 4.6. Statistical Analysis

Data analysis was performed using the SPSS statistical analysis software, version 17.0 (SPSS Inc., Chicago, IL, USA). The MARI of an isolate was defined as a/b, where a represents the number of antibiotics to which the isolate was resistant, while b represents the number of antibiotics for which the isolate was examined [17,38,39,45].

## 5. Conclusions

In the present study, we surveyed *K. oxytoca* contamination in 41 species of consumable aquatic animals sold in July, August, and September of 2018 and 2019 in Shanghai, China, including 21 species of mollusks, 17 species of fish, and 3 species of crustaceans. Among these, 40 species of the bacterium had not been detected previously.. *K. oxytoca* was for the first time isolated from 14 species, including 10 species of mollusks: *A. woodiana*, *B. areolata*, *C. cahayensis*, *H. rubra*, *M. antiquata*, *M. edulis*, *N. cumingi Crosse*, *S. subcrenata*, *S. constricta*, and *T. granosa*; 3 species of fish: *B. rock cod*, *C*. *auratus* (Crucian)*,* and *C. auratus* (Ditrema temmincki Bleeker); and one species of crustacean: *P. clarkii*. The other 27 species of aquatic animals were without *K. oxytoca*. Remarkably, *K. oxytoca* was found in high abundance in benthic animals, such as *N. cumingi crosse*, *T. granosa*, and *A. woodiana*.

None of *K. oxytoca* isolates (*n* = 125) harbored the toxin genes *mviM*, *tisB*, and *yqgB*. However, a high occurrence of virulence-associated genes was observed, including *brkB* (73.6%), *cdcB* (66.4%), *pduV* (64.8%), and *virk* (63.2%). Resistance to SXT (56.0%) was the most predominant among the isolates, followed by CHL (6.4%), TET (5.6%), and KAN (3.2%). Incidence of intermediate susceptibility to KAN (40.8%), and CIP (12.0%) were also observed. Approximately 8.0% of the isolates displayed MDR phenotypes. Meanwhile, high percentages of the isolates were tolerant to the heavy metals Cu^2+^ (84.8%), Pb^2+^ (80.8%), Cr^3+^ (66.4%), Zn^2+^ (66.4%), and Hg^2+^ (49.6%). Different virulence and resistance profiles were observed among the *K. oxytoca* isolates in 3 types and 14 species of aquatic animals. The ERIC-PCR-based genome fingerprinting of the 125 *K. oxytoca* isolates revealed 108 ERIC genotypes with 79 singletons, which demonstrated the considerable genetic diversity of the isolates.

Overall, the results of this study provided first experimental evidence for the presence of potentially virulent *K. oxytoca* in 14 species of commonly consumed aquatic animals, which poses a potential threat to food safety systems and the public health. In future research, the molecular mechanisms underlying the cross-resistance between antibiotics and heavy metals should be further examined in the emerging pathogen *K. oxytoca*.

## Data Availability

The raw sequences generated in this study are available from the NCBI GenBank under the accession numbers MZ380322 to MZ380328.

## Supplementary Materials

The following are available online at https://www.mdpi.com/article/10.3390/antibiotics10101235/s1, Table S1. The 41 species of aquatic animal products and the recovered *K. oxytoca* isolates, Table S2. Virulence-associated genes in representative *K. oxytoca* isolates, Table S3. Virulence-associated gene profiles of the 125 *K. oxytoca* isolates, Table S4. Tolerance of the 125 *K.* *oxytoca* isolates to eight heavy metals, Table S5. Oligonucleotide primers used in this study.

## References

[1] Singh L., Cariappa M.P., Kaur M. (2016). *Klebsiella oxytoca*: An emerging pathogen?. Med. J. Armed Forces India.

[2] Håkonsholm F., Hetland M.A.K., Svanevik C.S., Sundsfjord A., Lunestad B.T., Marathe N.P. (2020). Antibiotic sensitivity screening of *Klebsiella* spp. and *Raoultella* spp. isolated from marine bivalve molluscs reveal presence of CTX-M-Producing, *K. pneumoniae*. Microorganisms.

[3] Schneditz G., Rentner J., Roier S., Pletz J., Herzog K.A., Bücker R., Troeger H., Schild S., Weber H., Breinbauer R. (2014). Enterotoxicity of a nonribosomal peptide causes antibiotic-associated colitis. Proc. Natl. Acad. Sci. USA.

[4] Power J.T., Calder M.A. (1983). Pathogenic significance of *Klebsiella oxytoca* in acute respiratory tract infection. Thorax.

[5] Paveglio S., Ledala N., Rezaul K., Lin Q., Zhou Y., Provatas A.A., Bennett E., Lindberg T., Caimano M., Matson A.P. (2020). Cytotoxin-producing *Klebsiella oxytoca* in the preterm gut and its association with necrotizing enterocolitis. Emerg. Microbes Infect..

[6] Tse H., Gu Q., Sze K.H., Chu I.K., Kao R.Y., Lee K.C., Lam C.W., Yang D., Tai S.S., Ke Y. (2017). A tricyclic pyrrolobenzodiazepine produced by *Klebsiella oxytoca* is associated with cytotoxicity in antibiotic-associated hemorrhagic colitis. J. Biol. Chem..

[7] Disse S.C., Meyer S., Baghai-Arassi A. (2018). Sepsis-associated purpura fulminans due to Klebsiella Oxytoca. Dtsch. Arztebl. Int..

[8] Ullah S., Elbita O., Abdelghany M., Tahir H., Tuli P., Alkilani W.Z., Suri J. (2016). Klebsiella oxytoca endocarditis with complete heart block. J. Investig. Med. High Impact Case Rep..

[9] Decré D., Burghoffer B., Gautier V., Petit J.C., Arlet G. (2004). Outbreak of multi-resistant *Klebsiella oxytoca* involving strains with extended-spectrum β-lactamases and strains with extended-spectrum activity of the chromosomal β-lactamase. J. Antimicrob. Chemother..

[10] Gunduz S., Altun H.U. (2018). Antibiotic resistance patterns of urinary tract pathogens in Turkish children. Glob. Health Res. Policy.

[11] Alemayehu T., Asnake S., Tadesse B., Azerefegn E., Mitiku E., Agegnehu A., Nigussie N., Marian T., Desta M. (2021). Phenotypic detection of carbapenem-resistant gram-negative Bacilli from a clinical specimen in Sidama, Ethiopia: A cross-sectional study. Infect. Drug Resist..

[12] Mann B.C., Bezuidenhout J.J., Bezuidenhout C.C. (2019). Biocide resistant and antibiotic cross-resistant potential pathogens from sewage and river water from a wastewater treatment facility in the North-West, Potchefstroom, South Africa. Water Sci. Technol..

[13] Mehana E.E., Khafaga A.F., Elblehi S.S., El-Hack M.E.A., Naiel M.A.E., Bin-Jumah M., Othman S.I., Allam A.A. (2020). Biomonitoring of heavy metal pollution using acanthocephalans parasite in ecosystem: An updated overview. Animals.

[14] Zhang M., Sun X., Xu J. (2020). Heavy metal pollution in the East China Sea: A review. Mar. Pollut. Bull..

[15] Mahino F., Nazura U., Mobarak H.M. (2014). Heavy metal in aquatic ecosystem emphasizing its effect on tissue bioaccumulation and histopathology: A review. J. Environ. Sci. Technol..

[16] Su C., Chen L. (2020). Virulence, resistance, and genetic diversity of *Vibrio parahaemolyticus* recovered from commonly consumed aquatic products in Shanghai, China. Mar. Pollut. Bull..

[17] Chen D., Li X., Ni L., Xu D., Xu Y., Ding Y., Xie L., Chen L. (2021). First experimental evidence for the presence of potentially toxic *Vibrio cholerae* in snails, and virulence, cross-resistance and genetic diversity of the bacterium in 36 species of aquatic food animals. Antibiotics.

[18] Ni L., Chen D., Fu H., Xie Q., Lu Y., Wang X., Zhao Y., Chen L. (2021). Residual levels of antimicrobial agents and heavy metals in 41 species of commonly consumed aquatic products in Shanghai, China, and cumulative exposure risk to children and teenagers. Food Control.

[19] Alboghobeish H., Tahmourespour A., Doudi M. (2014). The study of Nickel Resistant Bacteria (NiRB) isolated from wastewaters polluted with different industrial sources. J. Environ. Health Sci. Eng..

[20] Herridge W.P., Shibu P., O’Shea J., Brook T.C., Hoyles L. (2020). Bacteriophages of *Klebsiella* spp., their diversity and potential therapeutic uses. J. Med. Microbiol..

[21] Medrano E.G., Forray M.M., Bell A.A. (2014). Complete Genome Sequence of a *Klebsiella pneumoniae* Strain Isolated from a Known Cotton Insect Boll Vector. Genome Announc..

[22] Weingarten R.A., Johnson R.C., Conlan S., Ramsburg A.M., Dekker J.P., Lau A.F., Khil P., Odom R.t., Deming C., Parl M. (2018). Genomic analysis of hospital plumbing reveals diverse reservoir of bacterial plasmids conferring carbapenem resistance. mBio.

[23] Zhang L., Zhong J., Liu H., Xin K., Chen C., Li Q., Wei Y., Wang Y., Chen F., Shen X. (2017). Complete genome sequence of the drought resistance-promoting endophyte *Klebsiella* sp. LTGPAF-6F. J. Biotechnol..

[24] Christensen S.K., Gerdes K. (2003). RelE toxins from bacteria and Archaea cleave mRNAs on translating ribosomes, which are rescued by tmRNA. Mol. Microbiol..

[25] Kawano M., Aravind L., Storz G. (2007). An antisense RNA controls synthesis of an SOS-induced toxin evolved from an antitoxin. Mol. Microbiol..

[26] Acar S., Bulut E., Stasiewicz M.J., Soyer Y. (2019). Genome analysis of antimicrobial resistance, virulence, and plasmid presence in Turkish *Salmonella* serovar Infantis isolates. Int. J. Food Microbiol..

[27] Xu Z., Liu Z., Soteyome T., Hua J., Zhang L., Yuan L., Ye Y., Cai Z., Yang L., Chen L. (2021). Impact of *pmrA* on *Cronobacter sakazakii* planktonic and biofilm cells: A comprehensive transcriptomic study. Food Microbiol..

[28] Edelmann D., Oberpaul M., Schäberle T.F., Berghoff B.A. (2021). Post-transcriptional deregulation of the tisB/istR-1 toxin-antitoxin system promotes SOS-independent persister formation in *Escherichia coli*. Environ. Microbiol. Rep..

[29] Fedhila S., Guillemet E., Nel P., Lereclus D. (2004). Characterization of two *Bacillus thuringiensis* genes identified by in vivo screening of virulence factors. Appl. Environ. Microbiol..

[30] Woolhouse M., Farrar J. (2014). Policy: An intergovernmental panel on antimicrobial resistance. Nature.

[31] Praveenkumarreddy Y., Akiba M., Guruge K.S., Balakrishna K., Vandana K.E., Kumar V. (2020). Occurrence of antimicrobial-resistant *Escherichia coli* in sewage treatment plants of South India. J. Water Sanit. Hyg. Dev..

[32] Anh H.Q., Le T.P.Q., Da N.Q., Lu X.X., Duong T.T., Garnier J., Rochelle-Newall E., Zhang S., Oh N.H., Oeurng C. (2021). Antibiotics in surface water of East and Southeast Asian countries: A focused review on contamination status, pollution sources, potential risks, and future perspectives. Sci. Total Environ..

[33] Li F., Chen L., Chen W., Bao Y., Zheng Y., Huang B., Mu Q., Wen D., Feng C. (2020). Antibiotics in coastal water and sediments of the East China Sea: Distribution, ecological risk assessment and indicators screening. Mar. Pollut. Bull..

[34] Meng L., Liu H., Lan T., Dong L., Hu H., Zhao S., Zhang Y., Zheng N., Wang J. (2020). Antibiotic resistance patterns of *Pseudomonas* spp. isolated from raw milk revealed by whole genome sequencing. Front. Microbiol..

[35] Maharjan A., Bhetwal A., Shakya S., Satyal D., Shah S., Joshi G., Khanal P.R., Parajuli N.P. (2018). Ugly bugs in healthy guts! Carriage of multidrug-resistant and ESBL-producing commensal Enterobacteriaceae in the intestine of healthy Nepalese adults. Infect. Drug Resist..

[36] Wang X., Cui L., Li J., Zhang C., Gao X., Fan B., Liu Z. (2021). Water quality criteria for the protection of human health of 15 toxic metals and their human risk in surface water, China. Environ. Pollut..

[37] Xiao H., Shahab A., Xi B., Chang Q., You S., Li J., Sun X., Huang H., Li X. (2021). Heavy metal pollution, ecological risk, spatial distribution, and source identification in sediments of the Lijiang River, China. Environ. Pollut..

[38] Xu M., Wu J., Chen L. (2019). Virulence, antimicrobial and heavy metal tolerance, and genetic diversity of *Vibrio cholerae* recovered from commonly consumed freshwater fish. Environ. Sc. Pollut. Res. Int..

[39] Fu H., Yu P., Liang W., Kan B., Peng X., Chen L. (2020). Virulence, resistance, and genomic fingerprint traits of *Vibrio cholerae* isolated from 12 species of aquatic products in Shanghai, China. Microb. Drug Resist..

[40] Le T.T.Y., Grabner D., Nachev M., Peijnenburg W., Hendriks A.J., Sures B. (2021). Modelling copper toxicokinetics in the zebra mussel, Dreissena polymorpha, under chronic exposures at various pH and sodium concentrations. Chemosphere.

[41] Dickinson A.W., Power A., Hansen M.G., Brandt K.K., Piliposian G., Appleby P., O’Nell P.O., Jones R.T., Sierocinski P., Koskella B. (2019). Heavy metal pollution and co-selection for antibiotic resistance: A microbial palaeontology approach. Environ. Int..

[42] Zhang J., Plowman J.E., Tian B., Clerens S., On S.L.W. (2020). An improved method for MALDI-TOF analysis of wine-associated yeasts. J. Microbiol. Methods.

[43] Weisburg W.G., Barns S.M., Pelletier D.A., Lane D.J. (1991). 16S ribosomal DNA amplification for phylogenetic study. J. Bacteriol..

[44] Meacham K.J., Zhang L., Foxman B., Bauer R.J., Marrs C.F. (2003). Evaluation of genotyping large numbers of *Escherichia coli* isolates by enterobacterial repetitive intergenic consensus-PCR. J. Clin. Microbiol..

[45] Rivera I.G., Chowdhury M.A., Huq A., Jacobs D., Martins M.T., Colwell R.R. (1995). Enterobacterial repetitive intergenic consensus sequences and the PCR to generate fingerprints of genomic DNAs from *Vibrio cholerae* O1, O139, and non-O1 strains. Appl. Environ. Microbiol..

