# Supplementary material for: First Experimental Evidence for the Presence of Potentially Virulent Klebsiella oxytoca in 14 Species of Commonly Consumed Aquatic Animals, and Phenotyping and Genotyping of K. oxytoca Isolates
Source: Antibiotics (Basel). 2021 Oct 11;10(10):1235. doi: 10.3390/antibiotics10101235 (PMC8532785; doi:10.3390/antibiotics10101235)
Supplement: Supplementary file 1 [file antibiotics-10-01235-s001.zip › antibiotics-1379295-supplementary.pdf]

Table S1. The 41 species of aquatic animal products and the recovered *K. oxytoca* isolates.

Type	Species	Common name	No. of isolates
Fish	*Aristichthys nobilis*	Variegated carp	0
	Blotchy rock cod	Grouper	9
	Carassius auratus	Crucian	3
	Carassius auratus	Ditrema temmincki Bleeker	2
	Channa argus	Snakeheaded fish	0
	Erythroculter ilishaeformis	Topmouth culter	0
	Plectorhynchus cinctus	Grunt	0
	Lctalurus punctatus	Channel catfish	0
	Lateolabrax japonicus	Perch	0
	Misgurnus anguillicaudatus	Loach	0
	Mylopharyngodon piceus	Black carp	0
	Nibea albiflora	Spotted maigre	0
	Parabramis pekinensis	Bream	0
	Pelteobagrus fulvidraco	Banded catfish	0
	Scophthalmus maximus	Turbot	0
	Siniperca chuatsi	Mandarin fish	0
	Sparus berda	Yellowfin snapper	0
Mollusks	*Anodonta woodiana*	Unionidae	21
	Antigona lamellaris	Diagonal clam	0
	Atrina pectinata	Comb pen shell	0
	Azumapecten farreri	Farrer's scallop	0
	Babylonia areolata	Cowrie shell	5
	Babylonia formosae habei	Ivory shell	0
	Cipangopaludina cahayensis	Mudsnail	1
	Haliotis rubra	Abalone	10
	Mactra antiquata	Mussel	1
	Mactra veneriformis	White clam seed	0

	Moerella iridescens	Sea-pumpkin seeds	0
	Mytilus edulis	Mussel	2
	Neptunea cumingi Crosse	Sea snail	31
	Ostrea gigas thunberg	Oyster	0
	Pseudocardium sachalinense	Arctic surf clam	0
	Saxidomus purpuratus	Sowerby	0
	Scapharca subcrenata	Ark shell	5
	Sinonovacula constricta	Razor clam	6
	Solen strictus	Razor clam	0
	Spisula solidissima	Golden scallop	0
	Tegillarca granosa	Blood cockle	28
Crustaceans	*Eriocheir sinensis*	Chinese mitten crab	0
	Penaeus vannamei	Whiteshrimp	0
	Procambarus clarkii	Crayfish	1

Table S2. Virulence-associated genes in representative *K. oxytoca* isolates.

Genes	*K. oxytoca* isolate	NCBI accession number
brkB	8-1-3-28	MZ380322
cdcB	8-1-3-31	MZ380323
pduV	8-1-3-29	MZ380324
relE	8-1-2-5	MZ380325
symE	8-1-3-31	MZ380326
vagC	8-1-3-31	MZ380327
virK	8-1-3-29	MZ380328

Table S3. Virulence-associated gene profiles of the 125 *K. oxytoca* isolates.

No. of genes	Genotype	No. of isolates

0	~	1
1	*brkB*$^+$	12
	virk$^+$	5
2	*brkB*$^+$/*virk*$^+$	13
	cdcB$^+$/*virk*$^+$	1
	brkB$^+$/*vagC*$^+$	1
	brkB$^+$/*relE*$^+$	1
	vagC$^+$/*virk*$^+$	1
	brkB$^+$/*cdcB*$^+$	3
3	*brkB*$^+$/*vagC*$^+$/*pduV*$^+$	1
	brkB$^+$/*cdcB*$^+$/*symE*$^+$	1
	brkB$^+$/*pduV*$^+$/*virk*$^+$	1
	vagC$^+$/*pduV*$^+$/*symE*$^+$	1
	brkB$^+$/*vagC*$^+$/*virk*$^+$	1
	cdcB$^+$/*pduV*$^+$/*virk*$^+$	3
	brkB$^+$/*relE*$^+$/*virk*$^+$	3
	brkB$^+$/*cdcB*$^+$/*pduV*$^+$	9
4	*brkB*$^+$/*cdcB*$^+$/*pduV*$^+$/*symE*$^+$	2
	cdcB$^+$/*pduV*$^+$/*vagC*$^+$/*symE*$^+$	1
	brkB$^+$/*cdcB*$^+$/*pduV*$^+$/*vagC*$^+$	1
	brkB$^+$/*cdcB*$^+$/*pduV*$^+$/*virk*$^+$	7
	cdcB$^+$/*pduV*$^+$/*symE*$^+$/*virk*$^+$	1
	cdcB$^+$/*pduV*$^+$/*relE*$^+$/*vagC*$^+$	1
	brkB$^+$/*pduV*$^+$/*symE*$^+$/*vagC*$^+$	1
	brkB$^+$/*cdcB*$^+$/*pduV*$^+$/*relE*$^+$	1
5	*brkB*$^+$/*cdcB*$^+$/*relE*$^+$/*symE*$^+$/*virk*$^+$	1
	cdcB$^+$/*pduV*$^+$/*relE*$^+$/*symE*$^+$/*virk*$^+$	14
	brkB$^+$/*cdcB*$^+$/*pduV*$^+$/*symE*$^+$/*virk*$^+$	2
	brkB$^+$/*cdcB*$^+$/*pduV*$^+$/*relE*$^+$/*symE*$^+$	4
	cdcB$^+$/*pduV*$^+$/*relE*$^+$/*symE*$^+$/*vagC*$^+$	1

	cdcB^{+}/*pduV*$^{+}$/*relE*$^{+}$/*vagC*$^{+}$/*virk*$^{+}$	2
	brkB^{+}/*cdcB*$^{+}$/*pduV*$^{+}$/*relE*$^{+}$/*virk*$^{+}$	3
	brkB^{+}/*cdcB*$^{+}$/*pduV*$^{+}$/*vagC*$^{+}$/*virk*$^{+}$	1
	brkB^{+}/*cdcB*$^{+}$/*pduV*$^{+}$/*relE*$^{+}$/*vagC*$^{+}$	2
	brkB^{+}/*cdcB*$^{+}$/*pduV*$^{+}$/*relE*$^{+}$/*symE*$^{+}$/*virk*$^{+}$	8
	brkB^{+}/*cdcB*$^{+}$/*pduV*$^{+}$/*relE*$^{+}$/*vagC*$^{+}$/*virk*$^{+}$	4
6	*brkB*$^{+}$/*cdcB*$^{+}$/*pduV*$^{+}$/*symE*$^{+}$/*vagC*$^{+}$/*virk*$^{+}$	1
	brkB^{+}/*cdcB*$^{+}$/*pduV*$^{+}$/*relE*$^{+}$/*symE*$^{+}$/*vagC*$^{+}$	2
	cdcB^{+}/*pduV*$^{+}$/*relE*$^{+}$/*symE*$^{+}$/*vagC*$^{+}$/*virk*$^{+}$	1
7	*brkB*$^{+}$/*cdcB*$^{+}$/*pduV*$^{+}$/*relE*$^{+}$/*symE*$^{+}$/*vagC*$^{+}$/*virk*$^{+}$	6

Table S4. Tolerance of the 125 *K. oxytoca* isolates to eight heavy metals.

Heavy metal	Number of isolates with a maximum observed MIC (μg/mL)											Resistance	
	**3.125**	**6.25**	**12.5**	**25**	**50**	**100**	**200**	**400**	**800**	**1600**	**3200**	**No.**	**(%)**
Cd^{2+}				1	4	34	74[a]	11	1			12	9.6%
Cr^{3+}							1	6	35[a]	48	35	83	66.4%
Cu^{2+}							1	18[a]	40	61	5	106	84.8%
Hg^{2+}	32	31[a]	36	21	5							62	49.6%
Mn^{2+}	1						1	12	35	62[a]	14	14	11.2%
Ni^{2+}							49	39	36[a]	1		1	0.8%
Pb^{2+}									[a]				

	1		3	6	14	55	46	101	80.8%
Zn^{2+}			a						
		2	40	71	12			83	66.4%

Table S5. Oligonucleotide primers used in this study.

Genes	Primer	Sequence(5′-3′)	Annealing temperature (°C)	Elongation time (min)	Amplicon size (bp)	Reference
16S rRNA	27-F	AGAGTTT-GATCCTGGCTCAG	52	2	1540	[43]
	1429-R	TACGGCTAC-CTTGTTAC-GAC				
tisB	*TisB*-F	AGGACTG-CATCAAGTAGTT	49	1	90	This study
	TisB-R	GAGTGGA-TATCGTT-GTTCT				
mviM	*MviM*-F	CGCCGCCGTCTTACTCTT	56.2	1	916	This study
	MviM-R	GCAT-ACTGGTGGTCATTTGGAT				
virk	*Virk*-F	GCCATTATCAGCCAC-TATCAGTTC	56	1	945	This study
	Virk-R	GCAGCCACAG				

brkB	*BrkB*-F	CGTCGTTTGGTCCG-TAACCAC-GATGC	56	1	860	This study
	BrkB-R	CAG-TAC-ACCCAGAC-GAACAAA				
vagC	*VagC*-F	GCATCATTCTGCGTCCCG	56	1	155	This study
	VagC-R	AGCTCAAACCGTCTTTCATCG				
yqgB	*YqgB*-F	ACTATCG-CAATCCGAAGA	48	1	146	This study
	YqgB-R	GGACTGTTTATGGGTTGTT				
relE	*RelE*-F	TATGCTCGCCGCAATGGT	55	1	345	This study
	RelE-R	CGA-GAAGGGTCAAATACAAA-GAAA				
cdcB	*CdcB*-F	GAT-TAC-AAACTTCTTCACCCAG	51	1	309	This study
	CdcB-R	GCG-TATCCTTAC-CTCCTTG				

symE	*SymE*-F	ATTTCTTCCTTATCGCTCTGC	55	1	288	This study
	SymE-R	GCGTCAC-CTGATTGTGGG				
pduV	*PduV*-F	AAAATCAACACGGGAGGC	55	1	152	This study
	PduV -R	CAGCGAATCACGCACCAT				
ERIC	ERIC1R	ATGTAA-GCTCCTGGGGATTCAC			-	[45]
	ERIC2	AAGTAAGTGACTGGGGTGAGCG				
